# Utility of CT Radiomics and Delta Radiomics for Survival Evaluation in Locally Advanced Nasopharyngeal Carcinoma with Concurrent Chemoradiotherapy

**DOI:** 10.3390/diagnostics14090941

**Published:** 2024-04-30

**Authors:** Yen-Cho Huang, Shih-Ming Huang, Jih-Hsiang Yeh, Tung-Chieh Chang, Din-Li Tsan, Chien-Yu Lin, Shu-Ju Tu

**Affiliations:** 1 Department of Radiation Oncology, Keelung Chang Gung Memorial Hospital, Keelung 20445, Taiwan; yenchohuang@hotmail.com (Y.-C.H.); djboyan@hotmail.com (S.-M.H.); s19104011@gmail.com (J.-H.Y.); 2 Department of Medical Imaging and Radiological Sciences, College of Medicine, Chang Gung University, Tao-Yuan 33302, Taiwan; 3 Department of Radiation Oncology, Linkuo Chang Gung Memorial Hospital, Tao-Yuan 33305, Taiwan; cgmhnog@gmail.com (T.-C.C.); vottale@gmail.com (D.-L.T.); 4 Particle Physics and Beam Delivery Core Laboratory, Linkou Chang Gung Memorial Hospital, Tao-Yuan 33305, Taiwan; 5 Department of Medical Imaging and Intervention, Linkou Chang Gung Memorial Hospital, Tao-Yuan 33305, Taiwan; 6 Department of Nuclear Medicine, Linkou Chang Gung Memorial Hospital, Tao-Yuan 33305, Taiwan

**Keywords:** radiomics, delta radiomics, nasopharyngeal carcinoma, Cox regression, clinical outcome

## Abstract

Background: A high incidence rate of nasopharyngeal carcinoma (NPC) has been observed in Southeast Asia compared to other parts of the world. Radiomics is a computational tool to predict outcomes and may be used as a prognostic biomarker for advanced NPC treated with concurrent chemoradiotherapy. Recently, radiomic analysis of the peripheral tumor microenvironment (TME), which is the region surrounding the gross tumor volume (GTV), has shown prognostic usefulness. In this study, not only was gross tumor volume (GTVt) analyzed but also tumor peripheral regions (GTVp) were explored in terms of the TME concept. Both radiomic features and delta radiomic features were analyzed using CT images acquired in a routine radiotherapy process. Methods: A total of 50 patients with NPC stages III, IVA, and IVB were enrolled between September 2004 and February 2014. Survival models were built using Cox regression with clinical factors (i.e., gender, age, overall stage, T stage, N stage, and treatment dose) and radiomic features. Radiomic features were extracted from GTVt and GTVp. GTVp was created surrounding GTVt for TME consideration. Furthermore, delta radiomics, which is the longitudinal change in quantitative radiomic features, was utilized for analysis. Finally, C-index values were computed using leave-one-out cross-validation (LOOCV) to evaluate the performances of all prognosis models. Results: Models were built for three different clinical outcomes, including overall survival (OS), local recurrence-free survival (LRFS), and progression-free survival (PFS). The range of the C-index in clinical factor models was (0.622, 0.729). All radiomics models, including delta radiomics models, were in the range of (0.718, 0.872). Among delta radiomics models, GTVt and GTVp were in the range of (0.833, 0.872) and (0.799, 0.834), respectively. Conclusions: Radiomic analysis on the proximal region surrounding the gross tumor volume of advanced NPC patients for survival outcome evaluation was investigated, and preliminary positive results were obtained. Radiomic models and delta radiomic models demonstrated performance that was either superior to or comparable with that of conventional clinical models.

## 1. Introduction

A high incidence rate of nasopharyngeal carcinoma (NPC), an epithelial carcinoma that occurs in the nasopharynx, has been observed in Southeast Asia compared to other parts of the world. In 2018, the mortality from NPC was 72,987 globally [1,2,3,4]. This high mortality rate emphasizes the significance and healthcare impact it has worldwide. Concurrent chemoradiotherapy is recommended for locally advanced NPC patients in treatment guidelines. This integrated approach to NPC treatment enhances the effectiveness of therapies and increases survival rates among patients. At present, TNM staging is used as a prognostic indicator for treatment outcome evaluation. However, very different clinical outcomes may occur in patients with the same clinical staging. Therefore, it is essential to evaluate the clinical outcomes and manage the treatment in an individualized manner [5,6].

In response to this challenge, the growing field of radiomic research has generated interest as a means of enhancing the assessment of treatment outcomes and prognosis in NPC. Beyond traditional imaging techniques, radiomics provides a comprehensive method to quantify different aspects of tumor characteristics, leading us to a better understanding of tumor behavior and response to treatment. In other words, diagnostic imaging staging is somehow limited in correctly correlating to treatment response or clinical outcomes. In order to quantify different tumor characteristics, radiomics has been studied and suggested as an alternative for treatment outcome evaluation and prognosis [7,8,9].

Radiomics is a statistical and computational approach to extracting quantitative features from medical images, and these features have been shown to be useful in disease prognosis and treatment outcome prediction [7,10,11,12]. This computational technique based on noninvasive imaging can be applied to compute imaging markers for treatment response assessment. Tunali et al. concluded that some radiomic features are correlated with tumor hypoxia and angiogenesis [13]. A number of retrospective studies found radiomic analysis to be superior to conventional imaging diagnosis [7,14,15]. Liu et al. reviewed and summarized the applications of radiomics in imaging diagnosis and oncological treatment [16]. The authors suggested that radiomic research holds great promise. Furthermore, quantitative imaging features extracted from tumor regions provide multiple dimensional information for classifying tumor malignancy and hence assisting oncologists in making clinical decisions [7].

Radiomics is expected to play essential roles in the management of NPC patients by leveraging advanced imaging techniques to extract a high volume of quantitative features from medical images such as CT, MRI, and PET scans. These roles include imaging quantification of tumor phenotypes, cancer diagnosis improvement, tumor treatment response assessment, and assessing the prognostic value of clinical outcome prediction. Furthermore, the realization of precision medicine for NPC patients can be achieved if we combine patient-specific data and radiomic imaging features.

Applications of building radiomics models for treatment outcome evaluation have been studied recently, and high accuracy of predictive evaluation has been demonstrated [17,18,19]. In radiotherapy departments, computed tomography (CT) scanners are routinely used for treatment planning. A pre-treatment CT set is acquired not only for the purpose positioning immobilizations of patients but also for dosimetry calculations. During the treatment course, a new during-treatment CT image set is acquired for the consideration of reducing field or adaptive treatment planning. This second CT image set provides additional and more recent information to physicians for evaluating response to the first half of the treatment. Consequently, the pre-treatment image and during-treatment image sets can both simultaneously be utilized in radiomic analysis for predicting treatment outcome without additional extra clinical workflow. Furthermore, delta radiomics from these two CT sets can be utilized at different time points, and this idea was illustrated and shown to be useful in a study by Fave et al. [20].

Delta radiomics refers to a branch of radiomics focused on the analysis of changes in radiomic features over time [20]. Specifically, delta radiomics involves comparing these features across different time points to assess changes that occur in response to treatment or disease progression [21,22,23]. This approach can provide insights into how the tumor is responding to treatment, potentially even before changes are visible to the naked eye or through standard imaging assessments. Delta radiomics could involve analyzing radiomic features of tumor images obtained at different stages (e.g., different time points) of the disease or treatment to retrospectively evaluate various clinical outcomes such as treatment response, recurrence, or survival. This approach can help in understanding the disease better, predicting outcomes more accurately, and potentially guiding personalized treatment strategies.

Radiomics studies typically involve extraction of imaging features from a single tumor volume, reflecting the tumor-centric perspective that is a key element of radiomic analysis. This tumor-centric perspective has been a frequently adopted approach in radiomic analysis. However, a proximal region of the tumor microenvironment (TME) which is a peripheral tumoral region surrounding the tumor boundary was proposed and reviewed by Balkwill et al. and Wang et al. [24,25]. The authors summarized cell functions in this TME region and their corresponding biological roles in cancer development relative to treatment response. Furthermore, Laplane et al. and Zhou et al. reviewed the clinical roles of TME and analyzed TME textures [26,27]. They have suggested that the TME component could be used for predictive biomarkers in tumorigenesis.

The peripheral tumor region refers to the surrounding areas of a tumor, often at the interface between the tumor and surrounding normal tissues [28]. This region can play a crucial role in the tumor’s expanding development, invasion, and metastasis, as it is the place where the tumor interacts with its immediate environment. The tumor microenvironment encompasses the environment around a tumor, including surrounding blood vessels, immune cells, fibroblasts, signaling molecules, and the extracellular matrix. The TME is not merely a passive backdrop for tumor growth but an active participant in cancer progression, influencing growth, spread, and response to therapies. It plays a critical role in many different aspects of tumor biology. Therefore, understanding the interactions between the peripheral tumor region and the TME is vital for developing effective cancer treatments.

**Figure 1:**
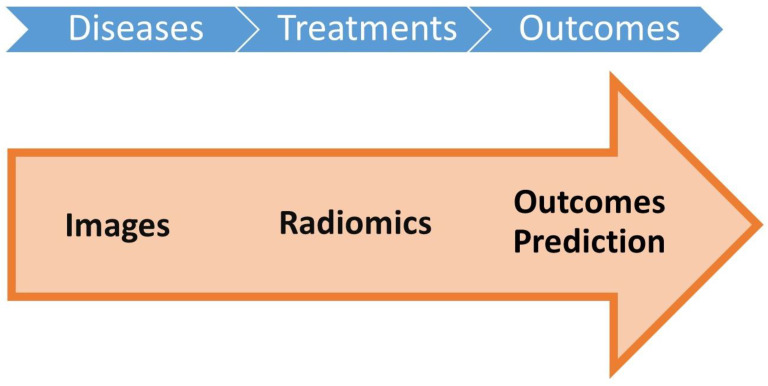
The workflow summary of radiomic application for clinical outcome prediction in this study.

The region of gross tumor volume (GTV) has been the target that is primarily focused on in radiation therapy. The potential usefulness of the peripheral tumoral microenvironment for the purpose of evaluating treatment response remains an unexplored area of radiation therapy. In this study, we aimed to evaluate locally advanced NPC outcomes after radiotherapy treatment by extracting radiomic features from the gross tumor peripheral tumor region (GTVp) which surrounds the GTV area. Additionally, we utilized delta radiomics and built various prognostic models with statistical model of Cox regression [20]. The C-index quantity was used for the performance evaluation of outcome prediction. A workflow illustration of radiomic analysis is shown in Figure 1.

## 2. Materials and Methods

### 2.1. Patient Characteristics

**Figure 2:**
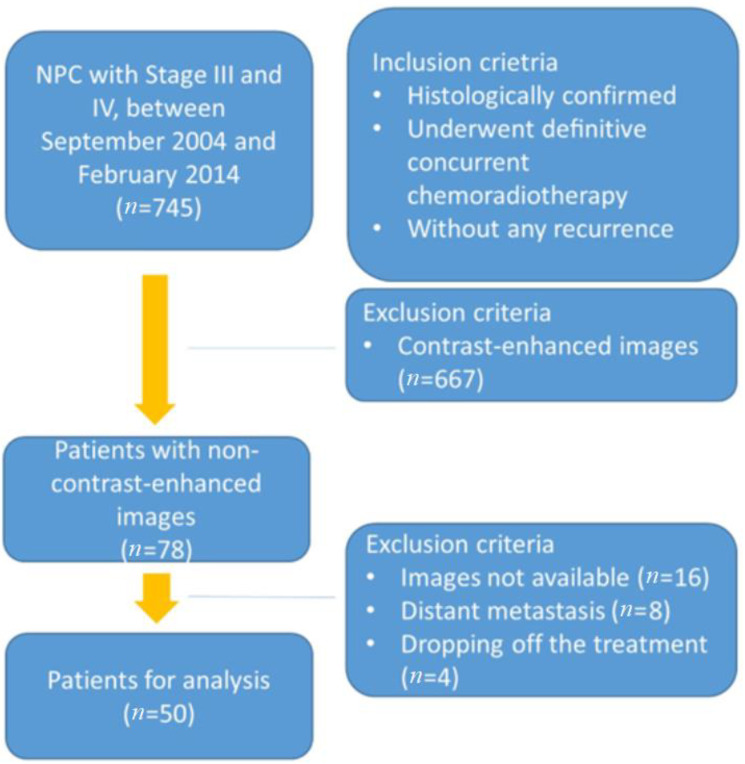
Patient selection criteria.

Between September 2004 and February 2014, a total of 50 patients with NPC stage III, IVA, and IVB (American Joint Committee on Cancer (AJCC) TNM staging system, 6th Edition) with standard radiotherapy treatment protocol were selected in this retrospective study (*n* = 50). The patient selection criteria are shown in Figure 2, and the characteristics are summarized in Table 1. The clinical factors include gender, age, overall stage, T stage, N stage, and treatment dose. All the advanced NPC patients were histologically confirmed and underwent definitive concurrent chemoradiotherapy with curative intent. No instances of recurrence, distant metastasis, or any other type of tumor were identified prior to the initiation of treatment. We excluded patients with stages I, II, IVC, or prior cancer history. Other exclusion criteria were as follows: dropping off the treatment, death within 90 days following the treatment, and failure to attend follow-up checkups. Overall, 21 patients died (42%), 5 patients developed a local recurrence (10%), and 6 patients developed distant metastases (12%) during the follow-up period. The median follow-up duration of the patients is a long period of 71.0 months (range: 5.2–159.9 months). This study is retrospective and was approved by the Institutional Review Board at Chang Gung Memorial Hospital (Number: 201801223B0). Given the retrospective design of this study, the requirement for obtaining informed consent was waived.

**Figure 3:**
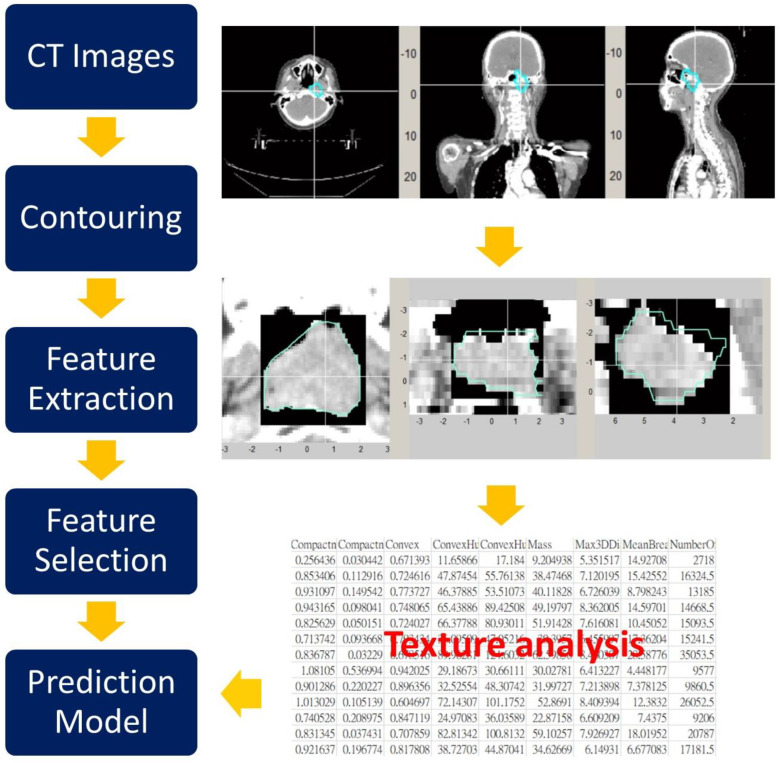
Illustration of extracting radiomic features and building the machine learning model.

A brief radiomic workflow for this work is presented in Figure 3. Non-contrast-enhanced CT images were obtained by two imaging scanners, GE HiSpeed and GE LightSpeed (GE Healthcare, Milwaukee, WI, USA). Both CT scanners used the same protocols and reconstruction method to minimize the variations in radiomic analysis between different scanners. Rachel et al. showed that using controlled protocol settings reduces variability in radiomic features [29]. The scanning parameters in this study were 120 kVp, current 150–398 mA, and CT slice thickness of 2.5 or 3.0 mm. Images were reconstructed to 512 × 512 pixels, which was around 0.98 mm × 0.98 mm for each pixel size.

### 2.2. Target Delineation and Segmentation

**Figure 4:**
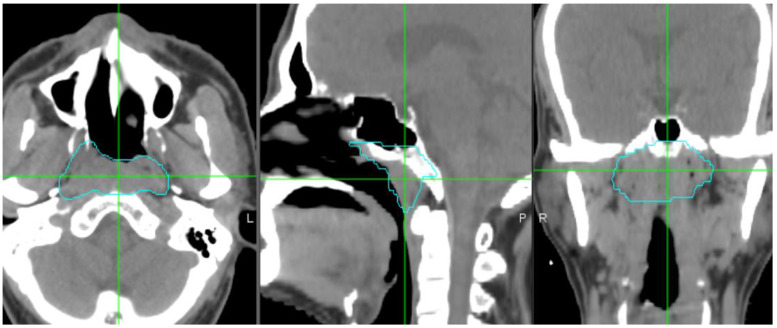
Contours of GTVt (cyan color) in the axial, sagittal, and coronal planes. The window of Hounsfield units (HUs) was set between −200 and 400 to remove air and bone area (water = 0 HU). “L” stands for left side, “P” stands for posterior, and “R” stands for right side.

**Figure 5:**
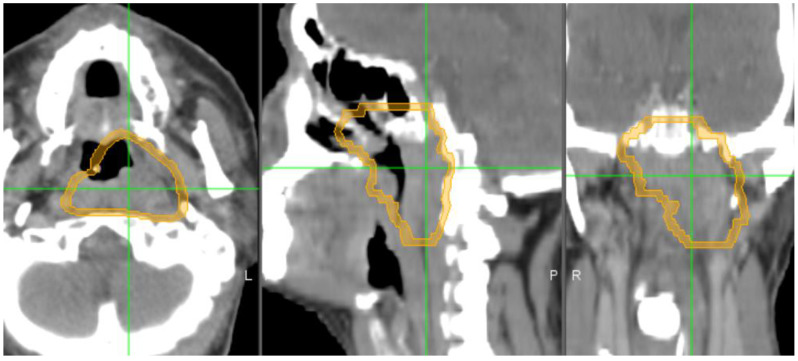
Segmentation of GTVp (i.e., tumor microenvironment showing in orange color). This peripheral region of tumor microenvironment is expanded 3 mm outside of GTV. “L” stands for left side, “P” stands for posterior, and “R” stands for right side.

For each patient, the segmentation of the NPC primary tumor was performed on the Eclipse V13.6 treatment planning system (Varian Medical Systems, Palo Alto, CA, USA). GTVt was contoured by our experienced radiation oncologists, as shown in Figure 4. A region of GTVp surrounding GTVt was created. In this study, GTVp is a proximal peripheral region which was between tumor edge of GTVt to 3 mm outer expansion [28,30], as shown in Figure 5. The choice of 3 mm dilation is based on our extended studies and observation of the results. There is no consensus on drawing the peripheral tumoral boundary [28]. Also, there is no agreement on segmenting the peripheral tumoral region, even on an institutional level. Nevertheless, this choice permits other groups to repeat and reproduce our work.

In radiation therapy, several regions are used to represent different geometric volumes of interest during treatment planning and delivery. Gross tumor volume, clinical target volume, and planning target volume are traditionally used for a medical physicist making a treatment plan. The gross tumor volume represents the visible extent of the tumor boundary as identified by a radiation oncologist. The clinical target volume extends beyond the visible tumor boundary to include areas that are suspected of harboring cancer cells but are not yet identifiable as true tumor mass on images such as CT images. This region takes into account the potential existence of tumor cells and their potential spreading territory. The planning target volume includes some extra expanding volumes for additional factors of potential treatment uncertainty and patient movement.

### 2.3. Radiomic and Delta Radiomic Features

An open radiomic software, IBEX (V1.0), was used for feature extraction [31]. Five categories of radiomics feature were used, including shape, gray-level run length matrix (GLRLM), intensity direct, neighbor intensity difference (NID), and gray-level co-occurrence matrix (GLCM). A total of 155 features were extracted using IBEX. In this study, the window of Hounsfield units (HUs) was set between −200 and 400 to remove air and bone area (water = 0 HU). A binning number of 256 and relative discretization were used in radiomic analysis [12]. The shape class applies mathematical equations to represent tumoral morphology. The GLRLM class calculates the length of consecutive pixels that have the same gray-level intensity in a specific direction. The intensity direct class calculates different types of statistical distribution in image grayscale levels. The NID class measures the variation in gray-level intensity between neighboring pixels or voxels in the matrix of a medical image. The GLCM matrix characterizes the texture of an image by calculating how often pairs of pixels with specific values and in a specified directional relationship occur in an image.

In addition to the pre-treatment CT planning (CT1), a second during-treatment CT planning (CT2) was obtained after (median of 23 days) the radiotherapy treatment was started. Radiomic features were extracted from both image sets. Delta radiomic features were obtained; from these, we subtracted CT1 feature quantities from those of CT2, and then, this quantity of CT2 minus CT1 was further divided by CT1 features for normalization, as shown in the following equation:

$$Delta\;Radiomics=\frac{CT2-CT1}{CT1}.$$

This approach enabled the quantification of longitudinal changes in radiomic features between the two time points (CT1 and CT2), capturing the potential evolution of tumor characteristics during the treatment course.

In this work, we studied three different results of clinical outcomes, including overall survival (OS), local recurrence-free survival (LRFS), and progression-free survival (PFS). Each outcome is dedicated to different types of clinical assessment. The length of time was calculated from the date of first treatment to that of the occurred event or the last follow-up date. A total of 15 models were included in this study.

### 2.4. Statistical Analysis

The SPSS (SPSS, version 17.0, SPSS/IBM, Chicago, IL, USA) statistical analysis software was used in this study. Cox regression was fitted for survival analysis by applying clinical factors and radiomic features. Multivariable classification via Wald forward selection method was applied for feature selection. All *p*-values of less than 0.05 were considered significant, and the best predictive model was built for each outcome. After models were built, the C-index was calculated using leave-one-out cross-validation (LOOCV) to evaluate each model’s performance. A C-index value of 1.0 suggests an ideal prediction [32].

## 3. Results

In total, 15 final prediction models were created using clinical factors and radiomics for three clinical outcomes: OS, LRFS, and PFS. The corresponding results of the C-index values are summarized in Table 2.

**Figure 6:**
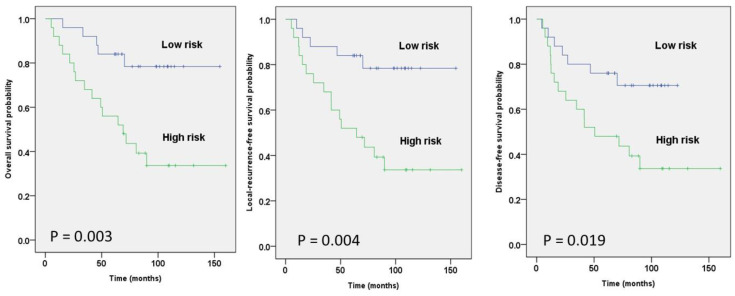
Kaplan–Meier survival curves from the clinical factor model analysis for three outcomes. Risks were stratified by clinical factors into two groups, and patients were dichotomized by the cut-off of risk in the survival curves.

In clinical models, age and overall stage were significant factors which were selected from the Cox regression of multivariate survival analysis. The selected radiomic features used in building models are summarized in Table 3. After models were built, the range of clinical factor models’ C-index was between 0.622 and 0.729. The risk prediction performance is illustrated in Figure 6.

**Figure 7:**
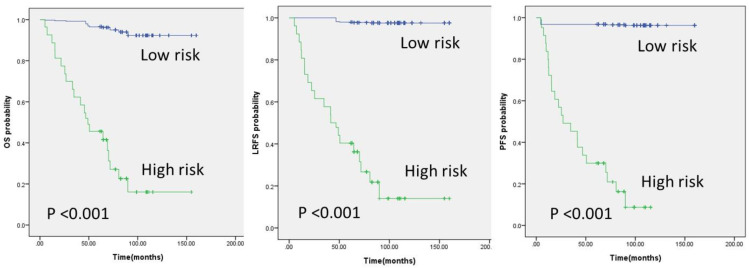
Kaplan–Meier survival curves from the GTVt delta radiomics model analysis for three outcomes.

**Figure 8:**
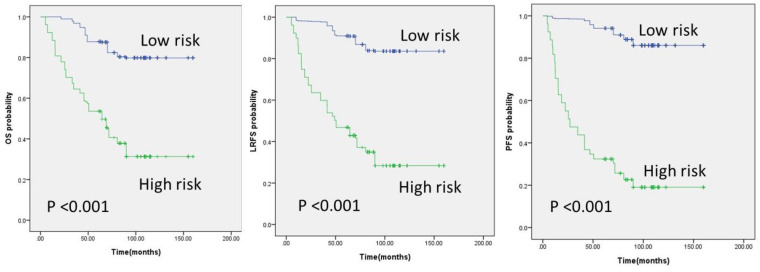
Kaplan–Meier survival curves from the GTVp delta radiomics model analysis for three outcomes.

In radiomic models, the range of C-index values was between 0.718 and 0.872. GTVt and GTVp models were in the range of 0.718 to 0.813 and 0.739 to 0.747, respectively. Among the delta radiomics study, GTVt delta yielded results in the range of 0.833 to 0.872, and GTVp delta yielded results in the range of 0.799 to 0.834. The risk prediction evaluation of the GTVt delta radiomics model and the GTVp delta radiomics model is illustrated in Figure 7 and Figure 8, respectively.

## 4. Discussion

Over the past few years, there has been significant exploration and development of radiomic prognosis models for various cancer sites in radiation therapy. Many clinical processes needed to be controlled before the application of these models in radiomics; these processes included imaging acquisition parameters, determining the use of either contrast-enhanced or non-contrast-enhanced images, the variations in tumor delineation, the binning size of CT gray levels, and image preprocessing influence. It is essential to clearly document these settings in radiomic research publications so results can be reproducible.

Traditionally, predicting clinical outcomes after treatment has relied on data from clinical factors such as patient age, disease stage, and comorbidity indexes. This study showed the promise of radiomic feature analysis in radiation therapy processes. To evaluate outcomes for advanced NPC patients after radiotherapy, radiomic features were extracted from CT images that were used in the treatment planning system. The results showed that outcomes prediction were comparable or superior to those obtained using clinical factors. Generally, GTVp radiomics models were superior to others, and the GTVt delta radiomics model showed higher C-index values than the others.

In Table 3, 2–10 significant features for each survival model are shown. In addition, important features which were selected in the OS, LLRS, and DFS models are ClusterTendendcy, Quantile975, Variance, Percentile80, and MaxProbability. Except ClusterTendendcy (Co-occurrence Matrix Features), they are all first-order radiomic features.

An important finding is that the delta radiomics method improved predictions more than analyzing only a single CT scan. The delta radiomics method is concerned with longitudinal change in quantitative radiomic features between the first time point of pre-treatment CT and the second time point of during-treatment CT images. Delta radiomics may provide additional information on tumor progression and treatment evaluation.

In most radiomic works related to tumor imaging characterization, the standard approach is delineating only the single tumor volume for feature extraction and analysis. Beyond the traditional approach, our study further analyzed the feasibility of using the tumor microenvironment (i.e., GTVp) for clinical outcome prediction. The peripheral regions of the TME may provide additional information regarding molecular characteristics for tumor classification. A similar result was found in the prostate in a study by Tu et al. [11]. In this study, GTVp is one of the frontier explorations, and the clinical role of TME was verified.

The images were acquired during a nearly 10-year period, and the performances under two CT scanners may be different. For this reason, beforehand, a quality assurance test comparing the two scanners was performed, and there was no significant difference in the correlation in radiomic features. The quality assurance procedures were based on the AAPM Task Group’s methodology for CT quality assurance and conducted by our certified medical physicist [33].

Delta radiomics refers to the concept of extracting and analyzing changes in radiomic features at different time points. Typically, these measurements are obtained before, during, or after a treatment course such as a series of radiation therapy procedures. These time-dependent points potentially allow us to apply radiomic features for monitoring and tracking disease progression over time. Delta radiomics can be used in several key areas, including treatment response evaluation, disease prognosis prediction, and early detection of any kind of treatment resistance.

In radiotherapy treatment planning, we define the clinical target volume’s boundary to include both the gross tumor volume and any areas that might contain microscopic malignant disease. This broader definition of CTV inherently recognizes the potential presence and influence of the tumor microenvironment in the spread and biological behavior of cancer cells.

The correlation between CTV and the tumor microenvironment in radiotherapy is essential, as the main purpose of CTV delineation is to include regions that may harbor microscopic disease which is influenced by the TME. The TME consists of different types of noncancerous cells. These cells include fibroblasts, immune cells, and endothelial cells. The biological functions of these cells are signaling molecules and the extracellular matrix. All these cells in the TME may contribute to further progression and metastasis of the tumor. Therefore, we are justified to consider extending beyond the GTV to include TME areas of potential microscopic spread in the image space.

The close relationship between the GTV and the tumor microenvironment in radiotherapy is a critical consideration for effectively irradiating not only the visible tumor but also the unseen factors that could influence clinical outcomes. This relationship underscores the need for a comprehensive approach to treatment planning that takes into account the complex biology of tumors and their surrounding environments.

One limitation of studies regarding the TME is the variation in the size and shape of the TME during dilation of the GTV in simulation CT images [28]. There is no consensus on drawing the peripheral tumoral boundary [28]. Also, there is no agreement regarding segmenting the peripheral tumoral region, even on an institutional level. Here, in our study, we performed dilation image processing by adding expanding pixels along the corresponding GTV boundary. This dilation procedure allows us to extract features from the TME, and it is repeatable for other groups carrying out similar research. Nevertheless, further research is necessary to determine the optimal TME range.

One challenge of radiomic analysis, in terms of determining the utility of contrast-enhanced images, is the issue of repeatability. The administration of contrast enhancement agent to patients, in general, is time-dependent. The procedures and settings of contrast agent administration are also dependent on the personal experience of the individual physician. Also, the outcomes of contrast agent administration are dependent on the physical conditions of patients. Therefore, CT images without the administration of contrast agent are likely to be more homogeneous across a population of patients.

One major purpose of our pilot study was to test the idea of the role of the peripheral tumor microenvironment (GTVp) in radiotherapy of advanced NPC patients. After the exclusion, a small number of patients remained. Under this circumstance, LOOCV was applied to lower the bias and make the statistical analysis more robust. It is important to note that our study was conducted on a cohort of 50 patients with advanced NPC (stages III, IVA, and IVB), which, while not a large sample size, is a relatively reasonable sample size for an exploratory study evaluating a novel radiomics approach. These 50 patients are all treated by the same radiation oncologist in our department, and contrast agent was not administrated to these patients. We acknowledge that further validation on larger patient cohorts is required to confirm and strengthen our findings. On the other hand, the large number of image features is a challenge, and it may cause a statistical type I error and an overfitting problem while applying Cox regression. To overcome this, a stepwise selection method was carried out to potentially remove the redundant features while performing fitting regression.

## 5. Conclusions

We investigated the role of the GTVp region in patients with advanced nasopharyngeal carcinoma. Radiomic analysis was utilized to evaluate survival in these patients, yielding preliminary positive outcomes. Our work tested the concept of delta radiomics by analyzing computed tomography images taken before treatment and during treatment at different time points. The study demonstrated that the delta radiomics approach and the prognostic models based on GTVp are valuable, showing predictive capabilities for survival assessment. However, additional validation and further research are necessary to confirm these findings and, thereby, explore their potential clinical implications.

## Figures and Tables

**Table 1:** General characteristic of selected patients of advanced nasopharyngeal carcinoma.

Patient Characteristics		
Gender	N	%
Male	38	76
Female	12	24
Age (years)		
Median	57.5	
Range	21.5–76.9	
Overall stage		
III	25	50
IVA	14	28
IVB	11	22
T stage		
T1	11	22
T2	4	8
T2a	2	4
T3	17	34
T4	16	32
N stage		
N0	2	4
N1	14	28
N2	23	46
N3	2	4
N3a	1	2
N3b	8	16
Treatment dose (Gy)		
Median	72	
Range	70–76	
Time between CT1 and CT2 (days) Median	23	

**Table 2:** Summary of C-index evaluation for different clinical outcome predictions.

	OS	LRFS	PFS
Clinical factors	0.729	0.654	0.622
GTVt model	OS	LRFS	PFS
CT1	0.720	0.813	0.718
Delta	0.843 *	0.833 *	0.872 *
GTVp model	OS	LRFS	PFS
CT1	0.739	0.747	0.741
Delta	0.799	0.808	0.834

OS: overall survival; LRFS: local recurrence-free survival; PFS: progression-free survival; GTVt: gross tumor volume—tumor; GTVp: gross tumor volume—peripheral tumor; *: the best model for each outcome evaluation.

**Table 3:** List of radiomic features selected as significant predictors in building the survival models. These features were identified through a rigorous statistical analysis using Cox proportional hazards modeling, highlighting their importance in predicting patient outcomes.

	OS	LRFS	PFS
GTVt	Compactness1	Kurtosis	Compactness1
	GlobalStd	ClusterTendendcy	MeanBreadth
	LocalStdMax		Orientation
	ClusterTendendcy		Quantile975
			ClusterTendendcy
			Correlation
GTVt Delta	LowGrayLevelRunEmpha	ConvexHullVolume3D	Convex
	LocalEntropyStd	RunPercentage	Roundness
	Quantile975	Quantile975	HighGrayLevelRunEmpha
	Coarseness	Coarseness	GlobalMin
	Contrast	AutoCorrelation	Quantile975
	Correlation	Variance	InverseDiffMomentNorm
	Dissimilarity		Variance
	SumAverage		
	Variance		
GTVp	Percentile80	Percentile80	Percentile80
	MaxProbability	MaxProbability	MaxProbability
			SumVariance
GTVp Delta	LocalEntropyStd	Convex	Orientation
	Percentile1	LocalRangeMean	LocalEntropyMax
	ClusterShade	LocalRangeMin	LocalEntropyStd
	ClusterTendendcy	LocalStdMin	LocalRangeMin
	Homogeneity2	Contrast	LocalStdMin
	InverseVariance	Correlation	Percentile50
	MaxProbability	DifferenceEntropy	Homogeneity2
		InverseVariance	MaxProbability
		MaxProbability	
		SumEntropy	

## Data Availability

All data in this study are available from the corresponding author upon reasonable request.

## Abbreviations

NPC: nasopharyngeal carcinoma; TME: tumor microenvironment; GTV: gross tumor volume; GTVt: gross tumor volume—tumor; GTVp: gross tumor volume—peripheral tumor; OS: overall survival; LRFS: local recurrence-free survival; PFS: progression-free survival.
